# Supplementary material for: Seminoma and Embryonal Carcinoma Footprints Identified by Analysis of Integrated Genome-Wide Epigenetic and Expression Profiles of Germ Cell Cancer Cell Lines
Source: PLoS One. 2014 Jun 2;9(6):e98330. doi: 10.1371/journal.pone.0098330 (PMC4041891; doi:10.1371/journal.pone.0098330)
Supplement: File S1 — ZIP file containing DMRforPairs output for significant regions. Please start from the html files. (ZIP) [file pone.0098330.s008.zip › figures/41249.pdf]

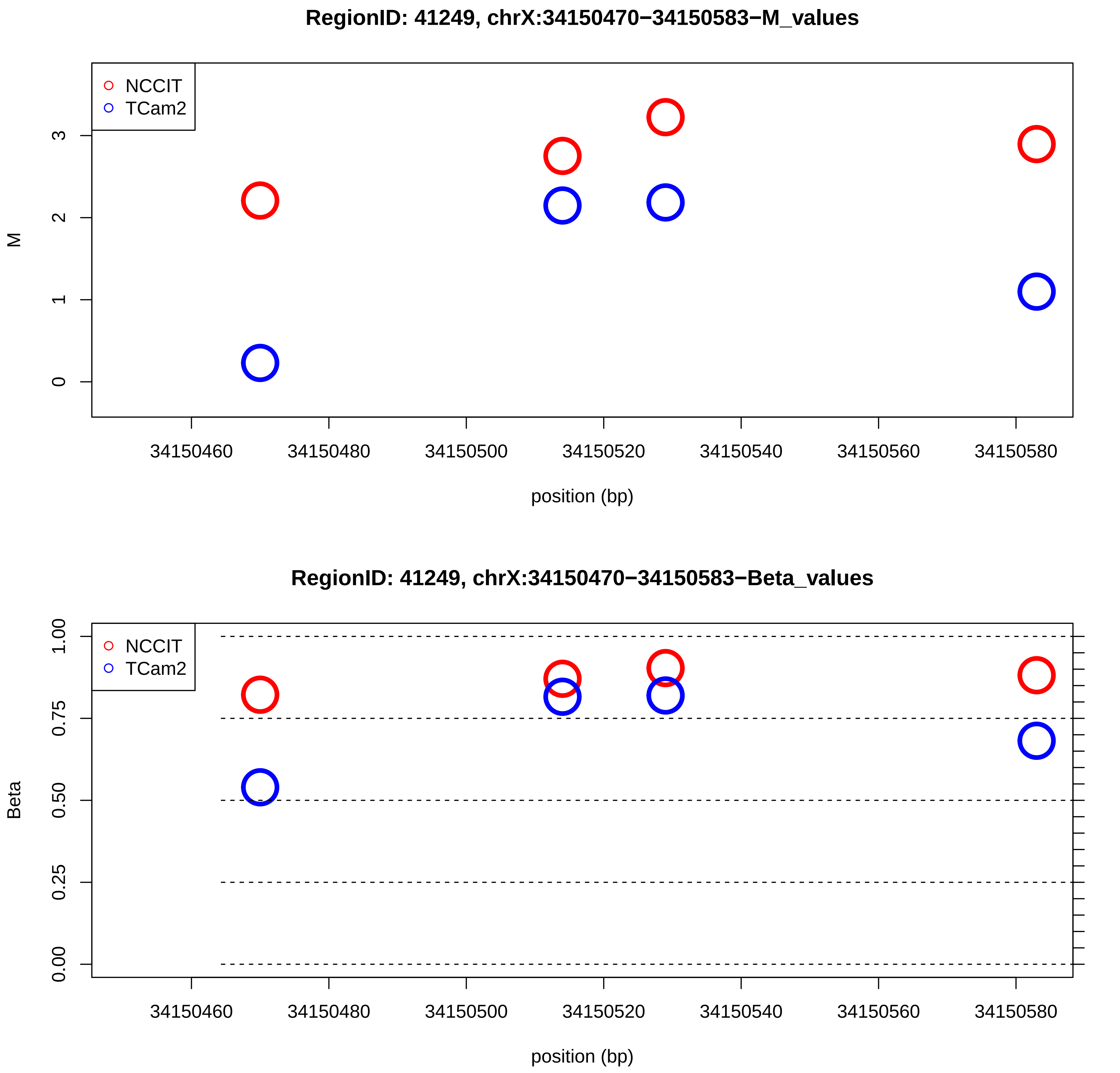
RegionID: 41249, chrX:34150470−34150583−M_values
NCCIT
TCam2
M
0
1
2
3
34150460
34150480
34150500
34150520
34150540
34150560
34150580
position (bp)
RegionID: 41249, chrX:34150470−34150583−Beta_values
NCCIT
TCam2
Beta
0.00
0.25
0.50
0.75
1.00
34150460
34150480
34150500
34150520
34150540
34150560
34150580
position (bp)
